# Evaluation of Bond Strength of Pressed and Layered Veneering Ceramics to Nickel-Chromium Alloy

**Published:** 2015-09

**Authors:** Mitra Farzin, Amir Alireza Khaledi, Behnam Malekpour, Mohammad Hassan Naseri

**Affiliations:** a Dept. of Prosthodontic, School of Dentistry, Shiraz University of Medical Sciences, Shiraz, Iran.; b Biomaterial Research Center, Dept. of Prosthodontic, School of Dentistry, Shiraz University of Medical Sciences, Shiraz, Iran.; c Postgraduate Student, Dept. of Orthodontic, School of Dentistry, Shiraz University of Medical Sciences, Shiraz, Iran.; d Dental Student, Student Research Committee, Dept. of Prosthodontic, School of Dentistry, Shiraz University of Medical Sciences, Shiraz, Iran.

**Keywords:** Metal, Bond, Pressable porcelain, Layering porcelain

## Abstract

**Statement of the Problem:**

The success of metal- ceramic- restorations (MCR) depends on the presence of strong bond between porcelain and metal substructure.

**Purpose:**

The purpose of this study was to evaluate the effect of hot pressing technique on the bond strength of a metal-porcelain composite in comparison to layering technique.

**Materials and Method:**

Thirty Nickel-Chromium specimens were produced by two methods; conventional porcelain layering on metal and hot pressing (n=15). Bond strengths of all specimens were assessed by the means of three–point bending test according to ISO 9693: 1999 (E) instructions. The data were analyzed using Students t-test (*p*< 0.001).

**Results:**

The mean ± SD bond strength of conventional and hot pressing technique was 48.29 ± 6.02 and 56.52 ± 4.97, respectively. Therefore, the conventional layering technique yielded significantly lower mean bond strength values than hot pressing technique (*p*< 0.001).

**Conclusion:**

This study showed that it is possible to improve metal–porcelain bond strength significantly by applying an overpressure during porcelain firing.

## Introduction

Metal ceramic restorations are used extensively in dental prosthetics.(1-2) In these restorations, aesthetic qualities of the ceramic materials can be used in combination with the strength and toughness of metal alloys to produce restorations that have both aesthetic and proper mechanical features.(3-7) Various types of alloys have been introduced for the metal ceramic restorations. Nobel alloys containing primarily gold palladium and a small percentage of indium have proven to be the most reliable ones. However, the major shortcoming of these alloys is their high cost and their lack of adaptability with various systems of ceramic. The alloys that are used as the base metal, enjoy certain desirable features such as low cost, increased strength, toughness, and greater resistance to distortion.However, they sometimes reveal additional oxide formations, prove difficult to finish and polish since they have a low ductility and exhibit a greater casting shrinkage.(8)

The presence of a strong bond between porcelain and metal substructure determines the success of the metal-ceramic restoration (M-CR).(9) It is also believed that the adhesion mechanism which exists between metal and porcelain is a micromechanical bond, which is compatible with the coefficient of thermal expansion (CTE), match vanderwals force and a proper metal oxidation and ion interdiffusion between metal and porcelain.(10-13)

Extensive studies have been carried out on the metal-porcelain bond.(14) Some effects were studied including firing cycle and temperature,(15-16) thermal and/or mechanical cycling,(17-18) cooling rate,(19) opaque layer thickness,(20) metal conditioners, adhesives.(21-22) However, the existence of various testing methods has limited the investigators’ ability in comparing the results of various M-C bond strengths. Therefore, although multiple mechanical tests have been carried out over the last two and a half decades, the more recent Schickerath three-point flexure test that has been standardized by the ISO FDIS 9693: 1999 (E) is now considered the gold standard for testing metal ceramic bond strength.(23)

Two methods are available for the application of porcelain on the metal core. The first method is the technique of layering. According to this traditional technique, first, opaque porcelain is applied on the core then dentin and enamel porcelains are used.(24) The second method is the technique of pressing. Following this technique, a complete contour wax up is applied on a core and a sprue is attached to it. The wax is eliminated in an oven and ceramics are pressed to the core under high temperatures.(25-26)

Comparative studies have been carried out on the bond strength of pressed ceramic to metal versus fusing feldspthic porcelain to metal by Venkatachalam *et al.*(26) and Schweitzer *et al.*(27) Although the pressable ceramic they used was indicated for all- ceramic restorations and not for metal- ceramic ones, they found no differences between the two techniques for the tested alloys. Unlike the mentioned studies, however, significant differences in metal-ceramic bond strength were experienced by some researchers(28-29) between hot pressing and traditional porcelain fusing to studied alloys.

According to the author´s knowledge, there was no published research regarding the comparison of porcelain application methods on bond strength of porcelains to nickel– chromium (Ni-Cr) alloys. Therefore, the current study aimed at measuring and comparing the bond strength of ceramics which were pressed or layered to a Ni-Cr alloy. The null hypothesis of the study was that the two mentioned techniques are equivalent in the bond strength. 

## Materials and Method

In this experimental study, pressed ceramic to core materials was selected as the experimental groups, and layer ceramics to the core materials was defined as the control groups (n = 15 per each group).

The features, composition, and the manufacturer of the materials which were used in this study are summarized in Table 1 and 2.

**Table 1:** The features and manufacturers of the used metal alloy

**Brand name**	**Composition**	**Manufacturer**	**0.2 yield point** ** (N/mm^2^) **	**Melting** **interval**	**Coefficient of thermal expansion** **(CTE)**
N E-Bond	Ni,Cr,Mo Si,Fe	Schutz Rosbach/Germany	550	1260-1350°C	14.1×10^-6^ k-1

**Table 2:** The features and manufacturers of the used porcelains

**Brand name**	**Composition**	**Manufacturer**	**Flexural strength** **(biaxial) [MPa]**	**Firing or Press temperature [°C]**	**CTE (100-500°C)** ** [10^-6^/K] **
IPS InLine (conventional metal-ceramic)	Feldspatic porcelain	Ivoclar Vivadent	80	900-930	13.2
IPS InLine (press-on-metal ceramic)			130	940-950	13.4

The preparation of specimen was performed within two stages; metal strip fabrication and ceramic veneering. In order to obtain Ni-Cr strips, stainless dies (25±1mm×3±0.1mm×0.5±0.05mm) were embedded in hard silicon rubber in accordance with ANSI/ADA specification NO.38 and ISO NO. 9693: 1999 (E)

**Figure 1:**
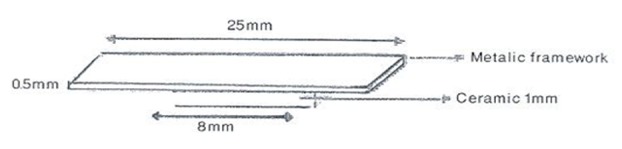
Final shape and dimensions of the Ceramic-Alloy specimen

Uniform thickness of molds was obtained as the result of pressing glass slide over the silicone material and die. The molds were filled with duralay acrylic resin (Pattern Resin; GC America, Alsip, IL) after removing the dies. Afterwards, rectangular acrylic templates were sprued and invested into phosphate bonded investment (Fujivest II; GC America, Alsip, IL) and then preheated according to the manufacturer's recommendation. Each alloy was casted by using natural gas and oxygen torch in a centrifugal casting machine (Multicast; DeguDent Hanau, Germany). After casting process, all molds were bench cooled. Carbide discs were used at low speed to remove sprues and separate metallic strips. Then, the metal strips were divested and cleaned by using airborne-particle abrasion. The surface of the specimens which would receive the ceramic was air borne-particle abraded once more by using 150-µm aluminum oxide particles at an angle of 45^◦^ for 10 seconds from nearly 2 centimeter distant and under pressure of 2 bars. The metal strips were cleaned by using a steam cleaner device, and were dried at the temperature of the room. Finally, the oxidation process was performed according to the instructions of the manufacturer. By using a graphite pencil, an area of 8×3 mm was marked on the metal strips and then the veneering ceramics were fired on the metal frames (Figure 1).

Proceeding to the process of veneering ceramics, the first and second opaque firings were performed for each group by applying the respective opaque following the manufacturer’s instruction.

Rectangular wax patterns (Prowax; Ivoclar, Vivadent, Liechtenstein) with approximately height of 1mm were fabricated on the opaque surface for the hot-pressed group. The sprues were attached to the top of the wax patterns and then the specimens were invested within the pressing ring and the lost wax technique was performed and ceramic ingots (Ivoclar, Vivadent, Liechtenstein) were pressed into the mold in the furnace (EP5000; Ivoclar, Vivadent, Liechtenstein) according to the manufacturer's instructions. For the layering group, a thin layer of the dentin porcelain was used in order to cover the opaque surface and was fired in the furnace in an oven (Vacumat 40; Vita Zahnfabrik, Bad Säckingen, Germany). Subsequently, the dentin porcelain was applied and fired twice. To maintain a uniform rectangular form for metal and veneer, excess ceramic was adjusted using a sintered diamond rotary instrument (Brasseler USA; Savannah, USA) and then the thickness was checked by digital dental caliper. After finishing the specimens, glaze firing was performed for all specimens. Afterward, three-point bending test was conducted on the specimens in a universal testing machine (model SS81, Instron) at a cross head speed of 1 mm/min. The bond strength (δ_b_) in MPa was determined by the following equation: δ_b_ = k × F(fail).

**Figure 2:**
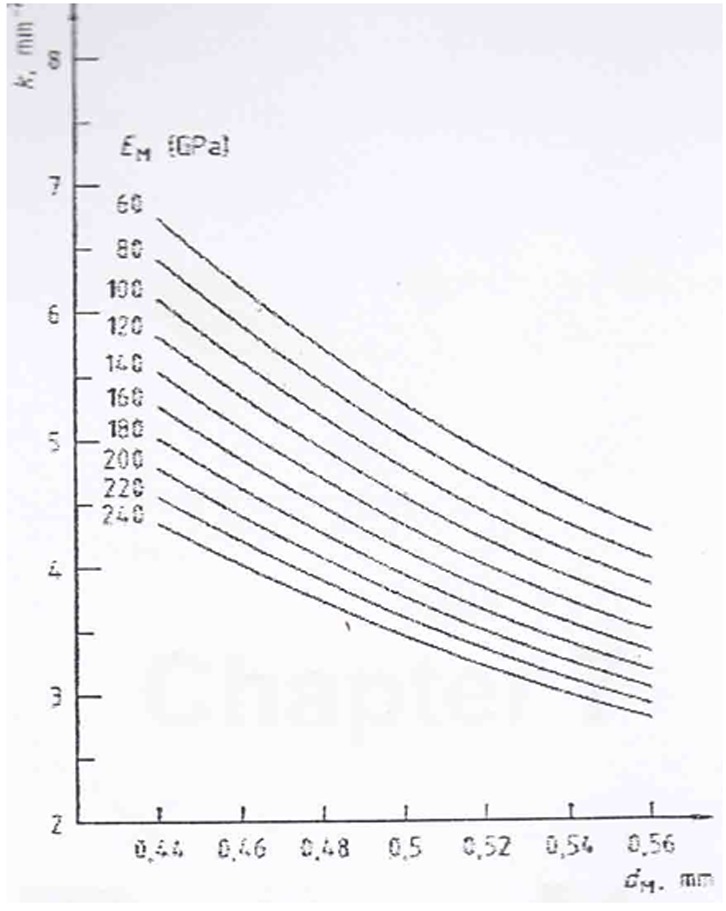
Diagram to determine the coefficient K as a function of metal substrate thickness dm and young's modulus E_M_ of the metallic materiel.

The coefficient K is a function of the thickness of the metal strip and the value of Young's modulus of the alloy which was derived from the diagram shown in the ISO 9693: 1999(E) (Figure 2). Statistical analysis was performed using SPSS 17. Students t- test was used to compare the mean tensile bond strength of the two groups.

## Results

Table 3 summarizes the tensile bond strength records for the two groups as mean±SD, minimum and maximum. The result (P-Value) of student´s t-test is also reported is this table. The result indicated that the conventional layering technique produced significantly lower mean bond strength measures (48.29 ± 6.02) than hot pressing technique (56.52 ± 4.97) (*p*< 0.001).

**Table 3:** The results of tensile bond strength measurement by three- point bending

	**Mean ± SD**	**Minimum**	**Maximum**	**p-value**
Layered	48.29±6.02	35.70	60.26	<0.001
Pressed	56.52±4.97	48.76	68.54	
Total	52.41±6.85	35.70	68.54	

## Discussion

The current study measured the effectiveness of the veneering technique on the bond strength of porcelains to metallic infrastructure cast in Ni-Cr alloy. On the basis of the findings of this study, the null hypothesis is rejected and the results indicated that metal ceramic bond strength is affected by veneering technique. However, the bond strength of the control and the experimental specimens ranged above 25 MPa, which is the minimum value determined in ISO 9693.(23)

Mechanical tests such as three- or four-point bending tests, biaxial flexural test, and shear test could be applied to compare or measure the bond strength between the ceramic and metal.(29-33) Studies which applied the shear test with different methods and various types of alloys revealed results ranging from 15 to 97 MPa.(3, 31-34) Since there is the lack of a universal methodology for measuring of the bond strength of metal porcelain systems, this range of variation is enormous. Therefore, the three-point flexure bond test suggested by the ISO guidelines as an international standard was employed for this study. If at least four out of six specimens have a debonding strength of more than 25 MPa, metal-ceramic systems enjoy enough qualification to pass ISO 9693.(23)

For complete fixed dental prostheses, metal-ceramic restorations have been a preferred restoration because of their long-term clinical use.(1-2,35) In order to establish an optimum esthetic outcome, veneering ceramics are classically layered on metal or zirconia core materials.(36-37) One possible technique is to press veneering ceramics to the core materials. Although the pressing technique itself is not considered as a recent technology, a process for pressing ceramics to metal and zirconia cores using the lost-wax technique and glass-ceramic ingots has been recently developed.(38)

The layering technique has been the principle method of the application of the veneering ceramics to the core materials. The layer is usually over built as a compensatory strategy to deal with condensation and firing shrinkage. In general, this technique requires good dexterity and multiple applications and firings. The failure rate caused by fracture and exfoliation of porcelain is 59.1% of the whole clinical failures despite the greater longevity of PFM restorations compared to all-ceramic restorations.(1-2,5) Therefore, there still is some work to do in the increasing of metal–porcelain bond. The pressing technique, suggests that a complete contour anatomical waxing is performed on a core, and subsequently, a sprue is attached to the wax and the wax-core complex invested. The wax is cleaned up in an oven and ceramics are heat-pressed into the mold and to the core; thereby, reproducing the anatomy created in the wax and allowing for the creation of the desired tooth anatomy. Moreover, the firing shrinkage experienced with the layering technique is minimized, resulting in a better fit of the porcelain margins to the abutments. Since there is a support from the investment, distortion of the metal may be reduced during veneering.(24-25,38) Also hot pressing encourages a full contact between metal and porcelain, enhancing the diffusion of the elements in the metal–porcelain interaction zone and resulting in an extremely good chemical bonding without any residual porosity and cracks.(39)

In this study, pairs of pressable and layering ceramics from the same manufacturer were selected to reduce the variables. Hence, the metal ceramic specimens in both groups had similar procedures performed in terms of the process of opaque application. However, application of veneering ceramics was done either by pressing or layering.

Similar to our research and due to an increased presence of uniformly distributed leucite phase, some studies have employed low-fusing, leucite-based pressable ceramics to metals.(26-27) These ceramics provide some advantages such as high compressive strengths and high flexural strength over traditional porcelains, due to an increased presence of uniformly distributed leucite phase.(39) The desired shape of the specimens for pressed ones was obtained with a single pressing procedure. However, in the process of layering specimens three applications and firings were used and subsequently adjustments were needed to achieve the definitive shape for bond strength testing. This was due to the firing shrinkage of the layering procedure, but this is adequately representative of the clinical practice for generating the restorations. A set of different factors, such as material composition and properties, firing temperatures, cooling rates, operator’s skill, porosities, and fabrication process might have an effect on the quality and strength of the bond between the core and the veneering materials.

The result of current study is consistent with the findings of the investigations carried out by Henriques *et al.*(40) and Ishibe *et al.*(38) although they evaluated the bond strength of pressed and layered ceramics to noble alloys. In contrast to our study, Venkatachalam *et al.*(26) and Schweitzer *et al.*(27) found no significant differences between the two techniques. This disagreement could be attributed to the difference between the metal and also the porcelain materials that have been used in their study and our research. In our study, the veneering porcelains were selected from the same manufacturer in order to reduce the study variables as much as possible. Therefore, not only CTEs of both porcelains were compatible with metal substructure(less than 1×10^-6^) but also opaque material and also its application method was the same for both groups. Moreover, they could not ascertain the effect that additional steps of divestment and sprue removal had on the debond strength values of pressed ceramic samples characterized by larger standard deviations (SD). Another significant aspect to consider in their studies was the CTE mismatch between pressed ceramic and metal, which was greater than 3×10^-6 o^C. It is recognized that CTEs differences of 1.7×10^-6^ ^o^C or greater between metal and porcelain could cause shear stresses at the interface which could result in a weak metal–ceramic bond or to its ultimate failure. To assure that porcelain is under compression at the interface, the metal’s CTE should be slightly higher than of the porcelain, but, perfectly their mismatch should not be greater than 1×10^-6 o^C.(5, 7, 13) However in our study the tested metal and porcelain materials were compatible in regard to CTE.(39)

In the future, studies evaluating the bond strength with different combinations of metals and veneering materials, and also the effect of mechanical and thermal cycling protocols are suggested. Furthermore, various types of methodologies to assess the bond strength might be deliberated. 

## Conclusion

Within the limitation of this *in vitro* study, it may be concluded that the tested metal-ceramic composites reveal sufficient bond strength for optimum clinical performance of the restorations. The bond strength of metals to porcelains could be improved by hot pressing technique.
